# On the Treatment of Chronic Dysentery

**Published:** 1873-09

**Authors:** Stephen H. Ward


### On the Treatment of Chronic Dysentery.

By Stephen H. Ward, M.D., F.R.C.P.

The first thing to be insisted upon is rest in bed, and in the recumbent position, in which the bowels are best kept quiet.

Diet stands next in importance to rest. That kind of diet should be ordered which gives least work to the alimentary canal, and which is most likely to be assimilated should the mesenteric glands be implicated, and which will send down to the large bowel a minimum amount of irritating waste material. Milk is the best form of nourishment in these cases; flour boiled with milk is a good combination; farinaceous articles of diet are also admissible. As a rule, the patients do better without alcoholic stimuli; but where there is much prostration these must be given.

It is important that an even temperature should be maintained in the bed-room or ward by night as well as by day. It had long been remarked that patients passing, say, twenty stools in twenty-four hours, would pass a large proportion of them in the night-time. The action of the skin, which it is desirable not to check, can be evenly maintained in bed. Dr. Ward has found the application of a broad flannel roller in some cases to do good by carrying out the indication of support and local surface-warmth. During the period of convalescene, flannel next the skin, and otherwise adequate clothing, are essential.

Special remedial agents render important service in the relief of various spmptoms. An occasional dose of opium at night, where there are irritability and restlessness, may be given, not to lock up the bowels, but with a view of procuring sleep. A dose of castor oil, guarded with laudanum, is often of service in bringing away scybalous fecal matter that has been retained, and caused griping and distress. For the tenesmus from which some patients suffer so much, an injection of starch and opium is the best remedy. The possibility of irritation being kept up by hemorrhoids must not be lost sight of. The severe and oft-repeated straining in the earlier stages of the disease gives rise at times to prolapsus ani, which in the more advanced stage may become a source of annoyance, and require surgical aid.

The complexion and course of chronic dysentery may be modified by the association of some special cachexia, as that of scurvy, ague, or tuberculosis. Where such exists the treatment will have to be modified. Where there are evidences of scorbutic taint, lime or lemon-juice must be given. It is here that the Bael fruit, which has enjoyed so much repute in India, will be found useful. If there be any old malarious influence at work, the symptoms will exhibit periodicity—the patients will perhaps be worse on alternate days, and then quinine will be the remedy. Where cough, hectic, etc., point to the tuberculous diathesis, cod-liver oil and tonics are indicated.—*Half-Yearlg Abstract.*

